# The Commonly Used Stabilizers for Phytochemical-Based Nanoparticles: Stabilization Effects, Mechanisms, and Applications

**DOI:** 10.3390/nu15183881

**Published:** 2023-09-06

**Authors:** Fang Zhou, Tiffany Peterson, Zhaoyang Fan, Shu Wang

**Affiliations:** 1 College of Health Solutions, Arizona State University, Phoenix, AZ 85004, USA; fzhou42@asu.edu; 2 College of Integrative Sciences and Arts, Arizona State University, Phoenix, AZ 85004, USA; tapete11@asu.edu; 3 School of Electrical, Computer and Energy Engineering, Arizona State University, Tempe, AZ 85281, USA; zhaoyang.fan@asu.edu

**Keywords:** nanoparticles, stabilizers, stability, mechanism, application

## Abstract

Phytochemicals, such as resveratrol, curcumin, and quercetin, have many benefits for health, but most of them have a low bioavailability due to their poor water solubility and stability, quick metabolism, and clearance, which restricts the scope of their potential applications. To overcome these issues, different types of nanoparticles (NPs), especially biocompatible and biodegradable NPs, have been developed. NPs can carry phytochemicals and increase their solubility, stability, target specificity, and oral bioavailability. However, NPs are prone to irreversible aggregation, which leads to NP instability and loss of functions. To remedy this shortcoming, stabilizers like polymers and surfactants are incorporated on NPs. Stabilizers not only increase the stability of NPs, but also improve their characteristics. The current review focused on discussing the state of the art in research on synthesizing phytochemical-based NPs and their commonly employed stabilizers. Furthermore, stabilizers in these NPs were also discussed in terms of their applications, effects, and underlying mechanisms. This review aimed to provide more references for developing stabilizers and NPs for future research.

## 1. Introduction

Many phytochemicals, such as resveratrol, quercetin, and curcumin, have a variety of pharmacological effects, including anti-inflammatory and anti-cancer activities. However, their application and development have been constrained, due to their low levels of aqueous solubility, stability, and bioavailability [1,2,3,4,5,6]. To attain efficacious blood levels, substantial quantities of phytochemicals must be administered; however, this augments the incidence of side effects and toxicity. Thus, there exists a pressing imperative for researchers to explore innovative methodologies that can more proficiently augment the solubility and bioavailability of phytochemicals. Biocompatible and biodegradable nanoparticles (NPs) can be employed to overcome these issues [7,8,9,10]. Phytochemicals can be loaded into NPs, subsequently resulting in improved solubility, stability, and bioavailability [11]. In addition, it has been reported that nano-sized phytochemical particles showed increased bioactivities mainly through prolonged circulation time, easy adherence to biological membranes followed by cellular uptake, and longer detention duration [10,12]. Nevertheless, NPs confront a challenge of instability arising from their large surface area and high free energy. Furthermore, NPs are susceptible to aggregation when the surface charge nears zero or when surface modifications are absent. This instability issue may result in aggregation or a burst of the NPs [13,14,15,16,17].

Incorporating stabilizers into NPs can overcome the instability issue of NPs. It has been reported that NPs can be stabilized by appropriate polymers or surfactants and manufactured using appropriate techniques [18,19]. When preparing NPs, some previous studies investigated and indicated that stabilizers, endowed with the capacity to hinder NP aggregation and avert bursts, assume a crucial role in enhancing the stability of NPs [20,21]. In this review, the effectiveness of stabilization effects of stabilizers, underlying mechanisms, and applications in phytochemical-based NPs were discussed. In addition, their long-term safety, limitations, and future directions in the NPs were also discussed [22,23].

## 2. Stabilizers for Phytochemical-Based NPs Preparation and Their Effects

Currently, nanotechnology has emerged as a prominent technique for drug delivery on the nanoscale, garnering considerable attention in the field of pharmaceutical sciences. This biphasic system consists of small particles dispersed in an aqueous medium. Hydrophobic phytochemicals may be loaded into the hydrophobic core of lipid NPs or emulsions, or hydrophobic compartments of other types of NPs. This leads to a significant enhancement in the solubility and stability of the hydrophobic phytochemicals [8,9,10]. In general, nanoencapsulation possesses the remarkable potential to significantly increase aqueous solubility, stability, and bioavailability. As a consequence, this augmentation engenders enhanced bioactivity while concurrently mitigating the adverse effects and toxicity. Furthermore, surface modifications using stabilizers can prolong the circulation time of NPs and enhance their stability. The commonly used stabilizers are chitosan (CS), caseinate (CN), saponins, cyclodextrin, polyethylene glycol (PEG), lentinan, glycosylated lactoferrin, trehalose, didodecyldimethyl ammonium bromide, polyvinyl alcohol, etc. [9,24,25,26,27,28,29,30,31]. The use of stabilizers as discussed below can significantly enhance the broader applications of these NPs.

### 2.1. CS and Its Derivatives

**Figure 1:**
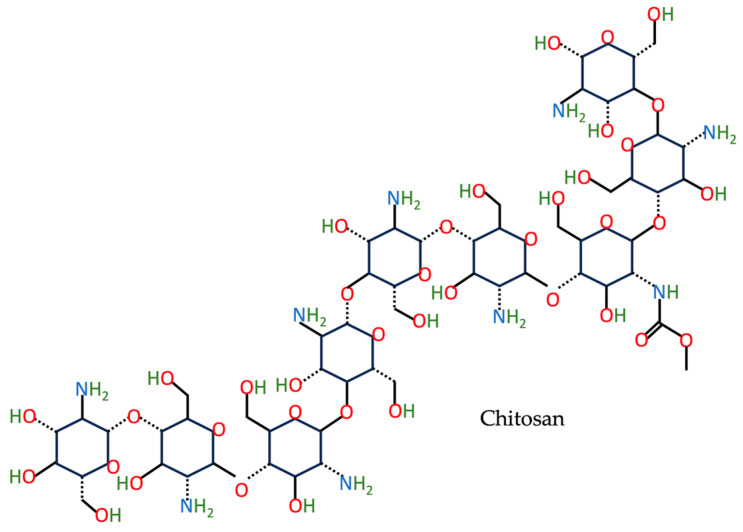
The chemical structure of CS based on the PubChem database [37].

The amphiphilic CS (Figure 1) is predominantly present in the exoskeleton of crustaceans and the cuticles of insects. It is constituted of d-glucosamine and *N*-acetyl-d-glucosamine, which are haphazardly dispersed and linked together via β-(1-4)-glycosidic bonds [32,33,34]. The synthesis of CS-incorporating NPs is an emerging application of CS, owing to its natural and renewable properties [35,36].

#### 2.1.1. Stabilizing Mechanisms

Prior studies suggested that CS, possessing robust biodegradability and biocompatibility, could be utilized as an NP stabilizer to enhance the morphology of NPs [38,39,40]. The primary amine groups of CS undergo protonation in the aqueous acidic environment, leading to the acquisition of a positive charge, which facilitates the effective encapsulation of negatively charged drugs through the electrostatic interaction [41,42]. In addition, due to its conformation in solution, it is a suitable option for stabilizing NPs [35,43].

Prior research employed CS as a stabilizer for silk-fibroin NPs [35]. Silk fibroin, a protein synthesized from the silk of silkworms, has gained significant attention in the biological and pharmacological fields due to its remarkable mechanical characteristics [44,45]. A major challenge associated with the utilization of silk-fibroin NPs in biomedicine is their tendency to aggregate in biological media due to ionic strength conditions. Researchers have primarily explored surface modification techniques involving the inclusion of CS and other stabilizers to overcome this issue [35]. Silk-fibroin NPs exhibit instability when the ionic strength of the dispersion is increased. However, in the aforementioned study, CS molecules encircled the silk-fibroin NPs mainly through electrostatic interactions, leading to the transformation of the lyophobic property of the colloidal particle into a lyophilic one [35]. In addition, a CS derivative, obtained through the ionic interaction between CS and oleic acid, has been harnessed to stabilize nanoemulsions of *Cymbopogon citratus*, commonly referred to as lemongrass. These nanoemulsions have consistently demonstrated colloidal stability within an aqueous milieu. The dimensions of the formulated nanoemulsions, employing concentrations of 0.05% and 0.1% CS, consistently uphold a size of around 300 nm throughout the entirety of the evaluation period [46]. This can be attributed mainly to the positive zeta potential of CS, which aids in the encirclement of the oil droplet’s shell by the CS shell through an electrostatic interaction [46].

In an earlier study, the naphthyl-grafted succinyl CS, a type of CS derivative, was employed as a stabilizer for the production of the andrographolide analogue NPs [8]. Andrographolide, a significant labdane diterpenoidal component of natural origin, has drawn considerable attention. Furthermore, andrographolide possesses various pharmacological properties, including antibacterial, anti-inflammatory, and anticancer effects [47]. In their previous research, the naphthyl-grafted succinyl CS used as polymeric micelles showed the greatest oral meloxicam delivery [48]. In their research, the andrographolide analogue NPs stabilized by naphthyl-grafted succinyl CS were well dispersed in aqueous media. The use of naphthyl-grafted succinyl CS as a stabilizer for andrographolide analogue NPs results in repulsion between each particle, creating an electrostatic environment. Additionally, it provides a steric effect, which further reduces the aggregation of these NPs. The results of the study also demonstrated that this formulation was physically and chemically stable for at least 6 months when stored at 4 °C [8].

To enhance electrostatic interactions and physical adsorption, other anionic compounds have been used with CS for coating NPs. Sanna et al. coated resveratrol-encapsulated poly(D, L-lactide-co-glycolide) (PLGA) NPs with cationic CS, and the properties of CS-coated PLGA-resveratrol NPs were investigated [49]. This interfacial phenomenon involves physical adsorption and/or electrostatic interactions, which were achieved by CS coating, and this research suggested that the electrostatic interactions were the predominant mechanism in improving the adsorption of CS on the surface of PLGA NPs [49,50]. 

#### 2.1.2. Applications

CS-coated (−)-epigallocatechin-3-gallate (EGCG)-loaded polymeric NPs (CS-EGCG-NPs) can treat psoriasis, an enduring inflammatory cutaneous ailment that may lead to a disrupted skin barrier function [51]. This study compared the therapeutic effectiveness of CS-EGCG-NPs and free-EGCG in the in vivo and in vitro models [51]. The findings revealed that compared to free EGCG, CS-EGCG-NPs treatment produced a four-fold superior efficacy in repressing inflammatory reactions in cultured human epidermal keratinocytes. Furthermore, topical application of CS-EGCG-NPs to lesioned skin considerably modulated mouse skin lesions and various psoriasis-associated inflammatory markers as compared to the administration of a high dose of free-EGCG, thus affirming the auspicious potential of topical delivery of EGCG via a CS-coated polymeric NP formulation.

CS and its derivatives, and many phytochemicals, have antimicrobial activity [52,53]. CS-coated phytochemical-encapsulated NPs might have increased antimicrobial activity. Our recent study indicated that CS-coated *trans*-resveratrol-encapsulated NPs inhibited the growth of *S. enteritidis*, *L. monocytogenes*, *E. coli*, and *S. aureus* as compared to *trans*-resveratrol-encapsulated NPs without CS, and further prolonged the shelf-life of strawberries after dipping into the NP solutions [54]. One aforementioned research implied that the presence of amino groups in CS could potentially facilitate the binding of lemongrass NP solution to the negatively charged bacterial surface, hence justifying its antimicrobial effects [46]. In that study, three *E. coli.* bacterial strains were utilized to evaluate the antimicrobial potential of the CS-coated lemongrass NPs. The results demonstrated a notable increase in antimicrobial efficacy against almost all the tested strains. Therefore, the integration of CS derivatives as a coating material in the lemongrass-NP solution not only conferred stability to the formulation but also enhanced its antimicrobial activity.

CS-coated phytochemical-encapsulated NPs have chemotherapeutic and anti-atherosclerotic properties for the management of cancer and atherosclerosis. The aforementioned CS derivative-stabilized andrographolide analogue NP solutions had higher anticancer activity and could also serve as a potential chemotherapeutic agent [8]. HCT116 human colorectal cancer cells were employed in that study, and the anticancer activity of CS derivative-stabilized andrographolide analogue NPs was assessed. The findings demonstrated that the groups treated with CS derivative-stabilized andrographolide analogue NPs exhibited elevated cytotoxicity. This outcome could be ascribed to the expanded surface area of the NPs, which presumably facilitated more cellular uptake. Small NPs have large specific surface areas as compared with their free forms under the same amount of mass, which could provide a larger interface between cells and NPs [55,56]. Our prior investigation also demonstrated that the nanostructured CS-coated EGCG reduced the cholesteryl ester content in macrophages and suppressed the levels of monocyte chemoattractant protein-1 in macrophages, thereby impeding the progression of atherosclerotic lesions [57].

A previous investigation also reported on the utilization of cationic CS- and alginate-coated PLGA NPs to enhance the physicochemical and pharmacokinetic characteristics of photosensitive compounds such as resveratrol, a natural polyphenol that possesses anti-inflammation, anti-obesity, and other benefits effects [58]. The results indicated that the NPs effectively attenuated the light-induced degradation of resveratrol, thereby prolonging its stability for a duration of six months [49]. Our previous research also indicated that resveratrol-encapsulated lipid nanocarriers and resveratrol-encapsulated liposomes exhibited lower degradation rates of resveratrol at 4, 22, and 37 °C as compared with free resveratrol under the light [59]. These outcomes of the nanosystem suggest that it could potentially be used for future chemotherapeutic purposes by enabling a sustained release of bioactive phytochemicals.

Previous research indicated that CS functioned not only as a stabilizer but also as a size-controlling agent in the synthesis of NPs [24,60]. By adjusting the amount of CS, the desired NP size can be readily achieved. This technique holds significant promise for the preparation of phytochemical-based NPs with specific sizes tailored to various practical applications.

### 2.2. CN

Casein, a ubiquitous protein present in milk, is a frequently employed emulsifying agent in the food sector [61,62]. Casein is frequently converted into CN, the majority sodium CN (Na-CN) [25]. The substitution of calcium salts with sodium salts results in the formation of the surfactant Na-CN, which serves as an efficient stabilizer, owing to its amphipathic micellar structure, heat stability, and colloidal ability [61,63].

One previous research has disclosed that the viscosity of Na-CN is primarily influenced by its concentrations and temperatures, rather than pH and ionic strength [20]. Elevated concentrations are particularly sensitive to temperature, as an upsurge in temperature causes a reduction in aggregation and viscosity. Additionally, the study suggested that subjecting Na-CN to temperatures up to 120 °C for a maximum of one hour led to a reduction in particle size of phytochemical-encapsulated NPs, thus promoting faster structural reorganization and phase separation [20]. This finding carries notable implications for CN-based NP formulations and their application, as the reduction in size and improved dispersibility would facilitate more effective drug delivery.

#### 2.2.1. Stabilizing Mechanisms

One study investigated the impact of Na-CN on the surface modification of shellac NPs loaded with quercetin [21]. Adding large amounts of quercetin directly into water-soluble foods is difficult due to its poor solubility and instability [64]. Shellac-NPs are a new delivery system, yet their utilization has been hindered by their high salt instability, poor redispersibility, and irreversible aggregation. To tackle these challenges, a protein modification method employing Na-CN has been implemented to enhance the stability of shellac NPs. Results have shown improved stability over time by encapsulating quercetin into Na-CN-coated NPs. Moreover, many hydroxyls (−OH), carboxyl (−COOH), and ammonium (−NH3) groups on Na-CN-coated quercetin NPs instigate the creation of hydrogen bonds, thereby assuming a pivotal function in fostering the adherence of Na-CN to the surface of shellac NPs. Furthermore, imperative roles in stabilizing NPs are fulfilled through hydrophobic and electrostatic interactions between Na-CN and shellac NPs. In particular, the hydrophobic interaction among hydrophobic quercetin, shellac, and the hydrophobic motif of Na-CN is the major stabilizing mechanism in this type of NPs [65]. Moreover, the protein matrix of Na-CN could entrap quercetin, thereby reducing its crystallinity. In addition, Na-CN as an electrostatic stabilizer has been utilized to improve the colloidal stability of quercetin and curcumin-encapsulated zein NPs [66,67]. Zein, a water-insoluble protein derived from corn, has been used to form zein NPs for the oral delivery of phytochemicals [68]. The CN coating layer conferred smaller particle size, enhanced redispersion, colloidal stability, and increased bioavailability of zein-quercetin NPs [66]. Additionally, Na-CN-stabilized zein-curcumin NPs exhibited favorable colloidal stability in the biopolymeric matrix and improved photostability against UV irradiation. Furthermore, Na-CN-stabilized zein-curcumin NPs were observed to remain stable under simulated gastrointestinal conditions [67].

Another study developed a quercetin-loaded zein NPs formulation using CN and CS-CN as stabilizers [69]. The findings demonstrated that CN-zein NPs exhibited a negative charge (due to consisting of a single negative-charged CN layer), while CS-CN-zein NPs (with a double layer of CS-CN coating) displayed a positive charge (due to the positive charge of CS). The research findings ascertained that the application of CS-CN and CN coatings markedly augmented the colloidal stability of zein NPs through the mechanisms of electrostatic repulsion, steric stabilization, or a synergistic combination thereof. Furthermore, it was observed that the CN layer surpassed the CS-CN shell in conferring superior colloidal stability.

#### 2.2.2. Applications

A previous study investigated the influence of Na-CN modification on quercetin-based NPs, and the results demonstrated that these NPs exhibited greater antioxidant activity than Na-CN and quercetin-based NPs [21]. The enhanced antioxidant activity of this nanosystem suggests the potential of Na-CN in delivering bioactive substances.

CN-coated zein NPs can remarkably increase the encapsulation efficiency of quercetin and curcumin, which can further enhance their delivery into the body [66,67,69]. It is noteworthy that both quercetin and curcumin are widely acknowledged for their beneficial bioactivities [3,70]. Moreover, since zein is a food-grade protein, it confirms the safety of the aforementioned nanosystems, as they are potentially ingestible.

### 2.3. Saponins

**Figure 2:**
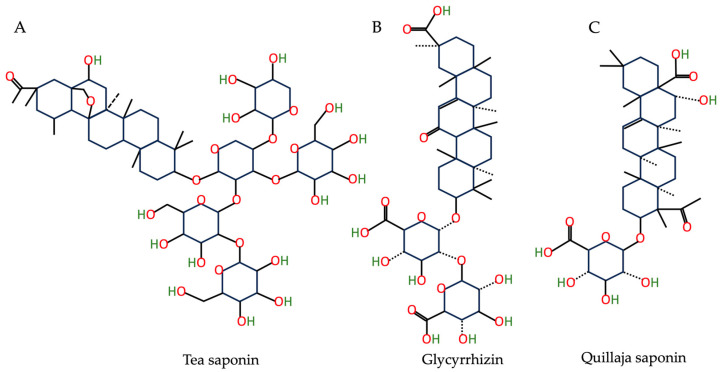
The chemical structures of (**A**) tea saponin, (**B**) glycyrrhizin, and (**C**) quillaja saponin based on the PubChem database [37].

Saponins, a diverse type of natural surfactant present in over 500 plant species, are currently being utilized as foam and emulsion stabilizers in nanosuspension and nano-emulsion fields [71,72,73]. These amphiphilic compounds feature a hydrophilic head and hydrophobic tail and may vary in terms of their aglycone head and the number of oligosaccharide tail chains [73], as illustrated in Figure 2. Moreover, they have demonstrated numerous health benefits including anti-inflammatory and anti-tumor activities [74,75].

#### 2.3.1. Stabilizing Mechanisms

One previous investigation has reported the use of tea saponins, a natural stabilizer, to stabilize NP solutions [26]. Tea saponins, extracted from the leaves of tea plants, are renowned for their inherent safety and environmentally friendly [76]. With their hydrophilic glycosyl and hydrophobic aglycons, tea saponins are potential emulsifiers in the creation and stabilization of NPs [77,78]. This study noted that tea saponins, even at low concentrations, have the ability to stabilize hesperidin NPs. This stabilizing effect may be attributed to the steric hindrance between NPs created by the non-ionic tea saponins. Furthermore, the hydrophilic glycosyl groups present in tea saponins can stretch and form a dense hydration film on NPs, thus bolstering the steric hindrance.

Another natural saponin, glycyrrhizin, was employed as a multifunctional stabilizer to form andrographolide-nanocrystal particles [79]. Andrographolide, known for its anti-inflammatory and antimicrobial properties, suffers from poor solubility and low bioavailability, which limits its potential medical applications [80,81]. The stabilization mechanism of glycyrrhizin arises from its interfacial characteristics and the influence of electrostatic forces. Some molecules of glycyrrhizin have the capacity to adhere to the surface of andrographolide nanocrystals, while others enable the entrapment of these nanocrystal particles within their intricate network structure, thereby curtailing their mobility.

One study reported that Panax notoginseng saponins exhibited efficacy in the stabilization of baicalein, a flavone characterized by limited solubility, and renowned for its diverse array of pharmacological impacts [82]. Unfortunately, its limited oral absorption has restricted its potential applications [83]. Results of this study indicated that baicalein nanocrystals are hydrophobic, and it was discovered that the Panax notoginseng saponins could readily adsorb onto their surface, preventing them from aggregating. The saponins also exhibited an electrostatic effect, which increased the dispersion stability and effectiveness of the NPs [84]. Quillaja saponin, a commonly used saponin emulsifier, is composed of two saccharide chains that assemble into a spherical micelle structure when immersed in aqueous solutions [72]. Research has demonstrated that NPs stabilized by quillaja saponin exhibit greater stability to flocculation and partial coalescence, which can be attributed to the saponin’s ability to increase steric and electrostatic repulsion between the lipid particles [72].

#### 2.3.2. Applications

Tea saponins and glycyrrhizin have been employed to stabilize hesperidin, andrographolide, and baicalein NPs, respectively [26,79]. Furthermore, Panax notoginseng saponins were also found to significantly reduce the aggregation of baicalein nanocrystals and further facilitate their delivery and therapeutic efficacy [84].

Furthermore, one previous research used food-grade quillaja saponin as the stabilizer to formulate NPs and hydrogenated vegetable oil was used as its lipid phase [72]. These findings have demonstrated that the promotion of lipid droplet crystallization was observed, and the temperature at which crystallization occurred decreased in tandem with an elevation in saponin concentration. An earlier study has indicated that the crystallization of the lipid phase can augment the stability of lipid droplets, subsequently fortifying the stability of the encapsulated bioactives [85]. These properties make quillaja saponin an excellent stabilizer for use in functional foods.

### 2.4. Cyclodextrin

**Figure 3:**
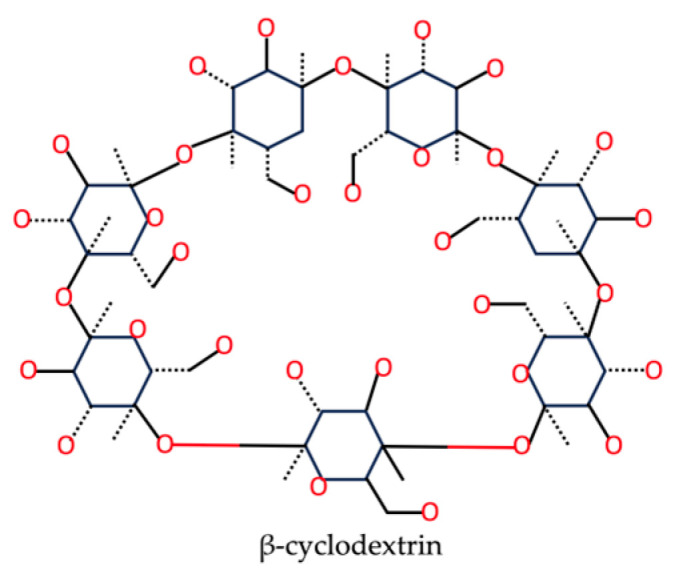
The chemical structure of β-cyclodextrin based on the PubChem database [37].

Cyclodextrin is a cyclic oligosaccharide (as illustrated in Figure 3 for β-cyclodextrin), which occurs naturally in starch. It has a hydrophilic exterior and a nonpolar, cone-shaped interior structure that is capable of encapsulating hydrophobic compounds in an aqueous solution, within its relatively more hydrophobic interior [86,87].

#### 2.4.1. Stabilizing Mechanisms

One previous study investigated the stabilizing effects of cyclodextrin on the rutin NPs [88]. Rutin, a polyphenolic flavonoid, has been widely used for its therapeutic potential [89]. In this study, cyclodextrin, Tween 80, and PEG-6000 were utilized and compared for their stabilizing abilities. The data underscored cyclodextrin as the paramount stabilizing agent for rutin NPs and rendered the smallest NP size. The stabilizing mechanism of cyclodextrin is attributed to the formation of electrostatic and protective layers that guard against particle aggregation and crystal growth [88,90]. Additionally, the hydrophilicity of cyclodextrin facilitated the solubility and stability of rutin NPs in water.

A study was conducted to investigate the impact of cyclodextrin as a water-soluble dispersion stabilizer on the oral bioavailability of silymarin NPs [91]. Silymarin, which is extracted from milk thistle, has been found to exhibit pharmacological activities, including antitumor and hepatoprotective effects [92,93]. Nevertheless, owing to its limited aqueous solubility, the bioavailability of silymarin following oral ingestion is hindered. In this study, the utilization of β-cyclodextrin was instrumental in impeding the aggregation tendency of silymarin NPs, wherein the mechanism of stabilization can potentially be ascribed to the saccharine structures inherent in β-cyclodextrin when dissolved in water. Moreover, the inclusion of β-cyclodextrin may also facilitate the generation of amorphous silymarin NPs during the high-pressure crystallization process.

Another study investigated the effect of 2-hydroxypropyl-β-cyclodextrin on modifying PLGA-paclitaxel NPs [94]. Effective delivery of paclitaxel in the body faces many difficulties and its overall therapeutic effect, such as anti-tumor activity, may be decreased [95,96]. The findings demonstrated that 2-hydroxypropyl-β-cyclodextrin reduced the size of PLGA-paclitaxel NPs and enhanced their stability. They explored the release profiles of paclitaxel from 2-hydroxypropyl-β-cyclodextrin/PLGA-paclitaxel NPs in 2% SDS solutions at three distinct pH levels (pH 7.4 to simulate the blood environment, pH 6.8 to imitate the extracellular microenvironment of tumors, and pH 5.0 to emulate the environment of late endosomes/lysosomes). The outcomes revealed that in contrast to unmodified PLGA NPs, the paclitaxel within the 2-hydroxypropyl-β-cyclodextrin/PLGA-paclitaxel NPs exhibited diminished drug release after a span of 72 h. Furthermore, the drug release of paclitaxel from the 2-hydroxypropyl-β-cyclodextrin/PLGA-paclitaxel NPs was found to be comparatively slower in comparison to the pH levels of 6.8 and 5.0. This alteration and mechanism of stabilization can be ascribed to the hydrophilic nature of 2-hydroxypropyl-β-cyclodextrin, which facilitated the establishment of an external layer around the NPs. In addition, some hydrophobic parts of PLGA were entrapped into the hydrophobic core of 2-hydroxypropyl-β-cyclodextrin, and the other uncoated hydrophobic PLGA parts formed an inner layer comprising a lipophilic nanostructure, effectively encapsulating the paclitaxel.

Sulfonate-β-cyclodextrin and CS have been used as fundamental constituents to synthesize NPs, capable of loading and regulating the release of berberine chloride [97], one phytochemical from traditional Chinese herbs, exhibits many beneficial effects, including antioxidant activity [98]. Optical transmittance assay of sulfonate-β-cyclodextrin-CS NPs at room temperature consistently demonstrated sustained optical clarity for a duration exceeding 7 h, underscoring the augmented stability of sulfonate-β-cyclodextrin-CS NPs. The enhanced stability of berberine chloride, facilitated by sulfonate-β-cyclodextrin and CS, can be attributed to the electrostatic interaction between the negatively charged sulfonate-β-cyclodextrin and the positively charged CS, promoting CS aggregation, and subsequently forming the protective multilayer assembly to encapsulate the drugs.

The hydroxypropyl-β-cyclodextrin has been used and combined with soybean lecithin to prepare annonaceous acetogenin (ACG) nanosuspensions [99]. ACGs are natural compounds possessing limited solubility and exhibit antitumor properties against various types of cancers [100,101]. The results indicated that hydroxypropyl-β-cyclodextrin-ACG NPs maintained their original size without aggregation for a duration of 12 h when exposed to simulated gastric or intestinal fluids. This finding also suggests that hydroxypropyl-β-cyclodextrin-ACG NPs hold the potential to be administrated orally. The stabilizing mechanism could be due to the architecture of hydroxypropyl-β-cyclodextrin, which is characterized by a hydrophilic exterior surface and a non-polar interior cavity [102]. Then, hydroxypropyl-β-cyclodextrin and soybean lecithin were self-assembled into an amphiphilic complex.

#### 2.4.2. Applications

Cyclodextrins hold substantial pharmaceutical potentials owing to their amphiphilic characteristics and resilience against degradation by human enzymes. Nevertheless, akin to other pharmaceutically encapsulated compounds enveloped by water-soluble surfactants, a rapid drug release rate also prevails [87,103]. Earlier research on cyclodextrin-stabilized rutin NPs demonstrated a significant improvement in drug dissolution rate. An in vivo rat paw edema inflammatory model was used, and the results showed that cyclodextrin-stabilized rutin NPs exhibited enhanced anti-inflammatory activity compared to free rutin. Furthermore, the data suggested that a greater amount of the drug permeated through mouse abdominal skin when using cyclodextrin-stabilized rutin NPs compared to the free drug [88]. This increased drug permeation could potentially enhance the efficacy of the anti-inflammatory activity.

As mentioned before, Zheng et al. synthesized 2-hydroxypropyl-β-cyclodextrin modified PLGA-paclitaxel NPs [94]. The in vitro A549 cells study revealed that compared to unmodified PLGA NPs, modified PLGA NPs significantly inhibited cell growth. These findings signify the potential utility of PLGA-paclitaxel NPs modified with 2-hydroxypropyl-β-cyclodextrin in the context of tumor treatment.

Chen et al. synthesized sulfonate-β-cyclodextrin-CS loaded curcumin NPs [97] and the data demonstrated that these NPs possessed the ability to disassemble under elevated pH conditions and regain their structure when the pH was lowered, aligning effectively with the contrasting pH environments of the stomach and intestines. This observation highlights the potential future utilization as a drug-delivery system.

CS and sulfonyl-ether-β-cyclodextrin-conjugated quercetin NPs have the potential as an effective anti-cancer approach for cervical cancer cells [104]. In that study, an in vitro cell viability assay was conducted to investigate the effects of this NP delivery system on HeLa cells. The half inhibitory concentration was assessed on Hela cells, and the findings demonstrated that quercetin-loaded NPs conjugated with CS and sulfonyl-ether-β-cyclodextrin exhibited the ability to reduce cell viability at lower concentrations of quercetin when compared to free-quercetin.

Hong et al. reported that hydroxypropyl-β-cyclodextrin stabilized ACGs NPs may improve the therapeutic anti-tumor efficacy of ACGs and indicate the potential oral application of ACG NPs [99]. Results indicated that hydroxypropyl-β-cyclodextrin-ACG NPs displayed minimal cytotoxicity against normal liver LO2 cell lines, while exhibiting significantly higher cytotoxicity against Hela and HepG2 cancer cell lines, indicating their potential application as anti-cancer agents. Additionally, the in vivo antitumor efficacy of ACG NPs was also investigated using H22-tumor-bearing mice. The data revealed that ACG NPs inhibited H22 tumor growth in a dose-dependent manner, with a high dose of ACG NPs (0.4 mg/kg) demonstrating limited tumor growth (2.03-fold increase). These results further emphasize the potential of ACG NPs in the development of anti-tumor drugs.

### 2.5. PEGs

**Figure 4:**
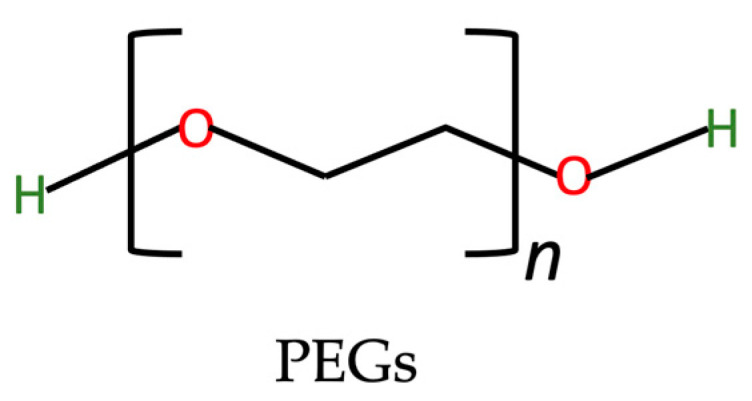
The chemical structure of PEGs based on the PubChem database [37,113].

PEGs (as illustrated in Figure 4) are polymers composed of ethoxy units and have been widely used in drug delivery systems due to their amphiphilic nature [105,106]. Additionally, PEG modification on NPs, known as PEGylation, has been reported to increase the NPs’ stability in vivo, thereby further enhancing their utility in drug delivery applications [107,108,109]. PEGs with diverse molecular weights exhibit distinct attributes. Specifically, the hydrophilic or amphiphilic nature of PEGs can undergo alterations in tandem with their molecular weight [110]. The molecular weight of the PEGs coating on the surface of NPs is a crucial factor that can influence the properties of the NPs in different ways [111,112].

#### 2.5.1. Stabilizing Mechanisms

Anthocyanins, which are flavonoids, are hydrophilic and unstable [114]. To enhance its bioavailability, researchers utilized PEG-2000 to coat PLGA-anthocyanin-loaded NPs as a delivery vehicle to improve the stability of the anthocyanin [114]. The findings showed a sustained release of anthocyanin in vitro, making it suitable for controlled delivery. This stabilization effect is believed to be attributed to the formation of a blood-compatible outer shell by PEG-2000, which modifies the surface of the PLGA-anthocyanin NPs.

One previous research designed the thymoquinone NPs based on the PLGA and PEG-5000 as the stabilizer [27]. Thymoquinone is extracted from black seed oil and has various health-promoting effects, such as anti-inflammatory and antioxidant activities, but is restricted by its low bioavailability [115,116]. Data exhibited extended release of thymoquinone capacity of PEG-5000-stabilized thymoquinone NPs as compared with free thymoquinone, with the peak release manifesting 15 to 30 h subsequently. Another study also employed PEG-5000 as the stabilizer and investigated its effects on the curcumin-loaded PLGA NPs formulation [117]. Curcumin is recognized to possess a variety of advantageous bioactivities [70], including antioxidant and anticancer properties [118], whereas its clinical applications are limited due to its low solubility and stability. Research suggests that PEG-stabilized and PLGA-based curcumin NPs manifest a more protracted half-life (roughly twice as long) in mice after 24 h monitoring, as opposed to curcumin alone, following intravenous administration of either curcumin or NPs [117]. These PEGs facilitate the formulation of PEG micelles via the surface coating of NPs, thereby engendering a protracted drug release mechanism.

#### 2.5.2. Applications

As previously noted, PLGA-anthocyanin NPs stabilized with PEG-2000 were formulated and their effects on the human neuroblastoma SH-SY5Y cell line were evaluated [114]. The findings indicated that these NPs hold promise as drug-delivery vehicles, with minimal cytotoxicity. Furthermore, the treatments involving these NPs were found to hinder the cell death triggered by Aβ_1–42_, which plays a crucial role in the progression of Alzheimer’s disease [119]. Moreover, PLGA-anthocyanin NPs stabilized with PEG-2000 were observed to attenuate the levels of proinflammatory cytokines and impede neurodegeneration in SH-SY5Y cells. This was evidenced by the reduced expression of tumor necrosis factor-alpha proteins and the modulation of caspase-3 levels. These observations highlight the safety and neuroprotective effects of PEG-2000 stabilized PLGA-anthocyanin NPs in the management of neurological disorders.

PEG-5000-stabilized thymoquinone NPs were found to diminish the levels of markers associated with cell proliferation and angiogenesis in human chronic myeloid leukemia cells. Moreover, anthocyanin NPs were observed to possess a superior capability to sensitize leukemic cells to apoptosis as compared to thymoquinone [27]. These findings suggested the potential use of PEG-5000-stabilized thymoquinone NPs in the field of anti-tumor therapy. Furthermore, PEG-5000-stabilized curcumin NPs were found to exhibit enhanced bioavailability compared to curcumin [117]. The findings additionally suggested that curcumin-loaded NPs stabilized with PEG-5000 exhibited heightened efficacy in the suppression of various transcription factors, notably including nuclear factor (NF)-kB, along with the restraint of its NF-kB-modulated proteins. This augmentation could potentially be attributed to the amplified cellular uptake of curcumin. The curtailment of NF-kB activation may be linked to the anti-tumor potential of PEG-5000-stabilized curcumin NPs. These observations further support the potential use of PEG-5000-stabilized curcumin NPs in the field of anti-tumor therapy.

### 2.6. Other Stabilizers for NPs

**Figure 5:**
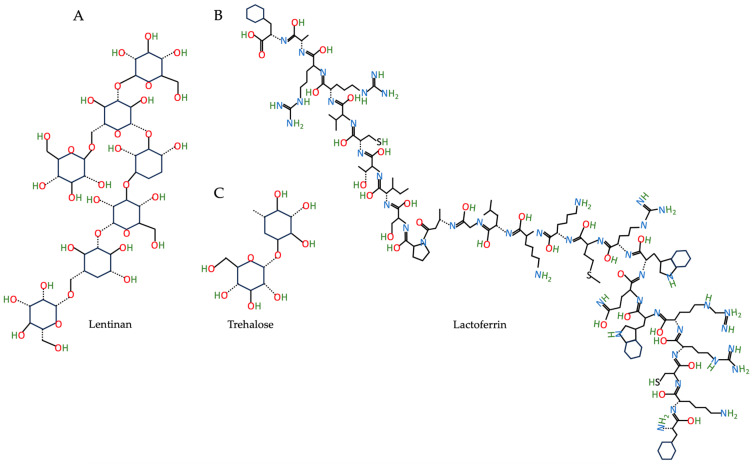
The chemical structure of lentinan (**A**), lactoferrin (**B**), and trehalose (**C**) based on the PubChem database [37].

There are many other stabilizers, such as lentinan, lactoferrin, trehalose, etc. (Figure 5), have also been used to stabilize phytochemicals-encapsulated NPs.

#### 2.6.1. Lentinan: Stabilizing Mechanisms and Applications

Lentinan is a natural β-1,3-D-glucan possessing diverse bioactivities, particularly its anticancer and immune-regulating properties [120,121]. Lentinan primarily induces immune responses in vivo to indirectly target cancer cells, and in vitro, it exhibits direct toxicity toward certain tumor cells [122,123]. As a polymer, lentinan has the potential to serve as a stabilizer in the formulation of drug-loaded NPs.

Previous research reported that lentinan, a polysaccharide found in shiitake mushrooms, is an excellent choice as an NP stabilizer due to its remarkable solubility resulting from the abundance of hydroxyl groups [121,124]. In another study, lentinan was employed as a natural stabilizing agent for the formulation of NP solutions containing regorafenib, an anticancer drug with limited aqueous solubility [28]. Results unveiled that lentinan induced a steric hindrance effect on the surface of regorafenib, consequently obstructing the aggregation of NPs. The regorafenib NPs enveloped by lentinan augmented the in vitro anticancer potency and oral bioavailability of regorafenib, concurrently mitigating its toxicity, as validated through rat investigations.

#### 2.6.2. Glycosylated Lactoferrin: Stabilizing Mechanisms and Applications

A prior investigation examined the storage stability of zein-7,8-dihydroxyflavone NPs stabilized using dextran-glycosylated lactoferrin via the Maillard reaction [29]. 7,8-dihydroxyflavone, a monomeric flavone compound, has been shown to alleviate brain-derived neurotrophic disorders, such as obesity and Alzheimer’s disease [125,126]. However, its application encountered limitations due to its metabolic processes within the intestinal tract and liver, resulting in a substantial diminution of its oral bioavailability [127]. Findings demonstrated that zein-glycosylated lactoferrin NPs exhibited enhanced stability when compared to zein-lactoferrin NPs. Furthermore, the aggregation of NPs was additionally inhibited due to the augmented electrostatic repulsion and steric exclusion provided by glycosylated lactoferrin. Moreover, the glycosylated lactoferrin created a protein-carbohydrate layer and produced the shielding effect, which bolstered the stability of NPs. Additionally, zein-glycosylated lactoferrin NPs displayed high encapsulation efficiency and proficient delivery of 7,8-dihydroxyflavone, implying that this nanosystem could be employed in dietary supplements and functional foods [29].

#### 2.6.3. Trehalose: Stabilizing Mechanisms and Applications

Trehalose functions as a disaccharide derived from glucose, employed by lower organisms as a strategy to endure the coldness of their natural habitats. Trehalose has been used as a non-toxic cryoprotectant, facilitating the cryopreservation of a wide array of biomacromolecules [128,129,130]. Furthermore, prior research has also demonstrated the potential of trehalose, as the cryoprotectant, in NP systems. Data revealed that CS-NPs subjected to lyophilization with cryoprotectants, including trehalose, reduced the average size and enhanced stability compared to those lyophilized without cryoprotectants. This improvement can be attributed to the facilitated redispersion of the NPs and the impeded agglomeration, achieved through the addition of cryoprotectants [30]. Additionally, Mandal et al., reported the utilization of polylactide (PL)-based biodegradable NPs loaded with anti-amyloidogenic catechin for the treatment of neurodegenerative disorders, and the PL is terminated with trehalose [131]. NPs forms of anti-amyloidogenic molecules, including trehalose, have shown potential superiority over their free forms [132]. Findings by Mandal et al., demonstrated the inhibitory effect of trehalose/poly(lactide)-catechin NPs on polyglutamine aggregation in HD150Q cells [131]. It is worth noting that the aggregation of polyglutamine may subsequently trigger the aggregation of other toxic proteins associated with neurodegenerative conditions, thereby impairing normal cellular function [131,133,134]. These observations indicate promising prospects for the future application of trehalose/PL-catechin NPs in the treatment of neurodegenerative diseases.

#### 2.6.4. Didodecyldimethyl Ammonium Bromide and Polyvinyl Alcohol: Stabilizing Mechanisms and Applications

Another investigation explored the impacts of ellagic acid encapsulated in PLGA- and polycaprolactone (PCL)-based NPs, stabilized by didodecyldimethyl ammonium bromide (DMAB) and polyvinyl alcohol (PVA) [31]. Ellagic acid, a potent dietary antioxidant, exhibited many pharmacological activities, particularly in the fields of cancer and diabetes [135]. However, its development has been impeded by its poor oral bioavailability. The findings of this investigation unveiled a substantial impact of the stabilizers on both the particle size and encapsulation efficiency of ellagic acid NPs. Notably, NPs stabilized with PVA exhibited larger particle size and higher encapsulation efficiency of ellagic acid, in contrast to those stabilized with DMAB. The uptake of ellagic acid in the intestine was higher in DMAB-stabilized NPs compared to PVA-stabilized NPs. These stabilizing effects could be attributed to the reduction of interfacial tension between the aqueous and organic phases by these stabilizers. Furthermore, DMAB-stabilized PLGA- and PCL-ellagic acid NPs were found to be more effective in mitigating cyclosporine A-induced nephrotoxicity in rats, further highlighting the potential therapeutic applications of these NPs in the treatment of various diseases.

## 3. Conclusions and Future Perspectives

Stabilizers play an important role in NP formation and function. Stabilizers including CS, CN, saponins, cyclodextrin, PEGs, lentinan, etc., can stabilize NPs mainly via the electrostatic interaction mechanism and outer layer formation. Hence, they can effectively deliver phytochemicals and further improve therapeutic efficacy by increasing phytochemicals’ encapsulation efficiency, loading capacity, and stability, and enhancing NP stability in body environments.

However, there are still some limitations in the field that need further investigation:

(1) The data gleaned from the aforementioned investigations postulate that the subsequent phase will entail pinpointing the particular molecular interplays between stabilizers and phytochemical-based NPs. A comprehensive understanding of the cellular uptake mechanisms necessitates a thorough investigation of NPs both with and without stabilizers, as well as their interaction with cells. It is recommended that forthcoming research encompasses comprehensive in vitro and in vivo assessments, as well as clinical trials, to evaluate the effectiveness of stabilizer-treated NPs.

(2) Notably, in NPs systems with stabilizer coatings [136], the utilization of NPs technology demonstrates a favorable influence on the prolongation of drug retention in the bloodstream, thus augmenting their therapeutic potential in NPs [10,12]. Despite some progress being made in earlier investigations, developing a coating that is more robust and stable, and provides NPs with the desired colloidal stability in biological environments, remains a daunting challenge.

(3) In order to achieve physiological compatibility between the phytochemicals and their respective stabilizers, future research must focus on optimizing the ratio of phytochemicals to stabilizers to determine the optimal NPs formulation.

(4) Whilst stabilizer-assisted nanomedicines have been thoroughly examined and have demonstrated exceptional qualities, a pertinent issue that demands attention is the safety of the ingredients utilized in the formulation. Stabilizers, such as surfactants, are considered to be potentially hazardous and allergenic substances. Overexposure to these substances, especially in substantial quantities or over prolonged periods, may have deleterious effects on one’s health [137,138]. As previously stated, different concentrations of CS can be employed as a size-control agent for the synthesis of the NPs [24]. However, the utilization of high concentrations of CS may have unforeseen consequences that warrant further consideration and investigation. Moreover, further studies pertaining to the size-modulating impacts of CS on phytochemical-based NPs are required in the future.

## Data Availability

Not applicable.
